# Hypoglycaemia in a Patient Taking Tetracyclines

**DOI:** 10.7759/cureus.45406

**Published:** 2023-09-17

**Authors:** Muhammad Fahad, Ryan Goindoo

**Affiliations:** 1 Internal Medicine, Peterborough City Hospital, Peterborough, GBR; 2 Diabetes and Endocrinology, Peterborough City Hospital, Peterborough, GBR

**Keywords:** oral doxycycline, rare side effect, acne vulgaris, tetracycline, hypoglycaemia

## Abstract

Hypoglycaemia with tetracycline use is a very rare and unknown side effect of the antibiotic. The case highlights the importance of recognizing and managing this potential adverse effect. We present a case of an adolescent male with acne vulgaris who developed hypoglycaemic episodes after initiating tetracycline treatment and was referred by his General Practitioner to the Endocrinology clinic. The hypoglycaemia symptoms settled once tetracyclines were stopped. Acne vulgaris is a prevalent skin condition among adolescents, and antibiotics such as doxycycline and tetracycline are commonly used to treat severe cases of acne. While generally well-tolerated, rare side effects on glucose metabolism have been reported. Antibiotics are not well known to cause hypoglycaemic spells on their own. It is mostly when these antibiotics are started in patients taking other regular medications that the interaction between these medications causes hypoglycaemia.

## Introduction

Acne vulgaris is a common skin condition affecting many adolescents worldwide [1]. Although it is typically benign, pharmacological interventions are sometimes necessary and tetracycline is a treatment of choice for moderate-to-severe acne [2]. While generally well-tolerated, like any medication, tetracycline carries the risk of adverse effects, including rare reports of hypoglycaemia associated with its use [3-5].

Tetracyclines, due to their broad-spectrum efficacy and relatively favourable safety profile, have been a mainstay option for various bacterial infections. However, recent clinical observations of their potential to induce hypoglycaemia, particularly in adolescent patients, have raised questions about their safety.

There is limited literature available on this subject and the exact mechanism underpinning the connection between tetracycline use and hypoglycaemia is still unknown. A comprehensive understanding of the pathophysiology involved is yet to be achieved. With this case report, we aim to contribute to the ongoing discourse by presenting a case where the temporal relationship between tetracycline treatment initiation and recurrent hypoglycaemic episodes, followed by symptom resolution upon discontinuation, strongly implies a causal relationship.

## Case presentation

We describe the case of a non-diabetic adolescent male presenting with a two-year history of acne vulgaris. He presented to his General Practitioner (GP) for acne vulgaris and was started on treatment with doxycycline and adapalene/benzoyl peroxide. He continued this for one month and after a month of poor response to oral doxycycline 100 mg daily and topical adapalene/benzoyl peroxide, he was switched to oral tetracycline with the same topical treatment with adapalene/benzoyl peroxide which continued for three months. He had not been on any other regular medications during this period.

Approximately two months into the treatment with tetracyclines, he started experiencing episodes of a ‘bubble’-like sensation in his chest, each episode lasting for 5-10 minutes and then resolving spontaneously. He also experienced dizziness sometimes. He denied taking any other medications or supplements. These episodes persisted for four weeks until he sought medical attention, leading to the diagnosis of recurrent hypoglycaemia with his GP who tested the post-prandial blood glucose level which was 3.2 mmol/L and the repeat post-prandial level was 3.1 mmol/L with an HbA1c of 33 mmol/mol. He was subsequently referred to endocrinology by his GP for a workup of recurrent hypoglycaemia. The endocrinologist stopped the tetracyclines and his symptoms settled, and he never experienced symptoms thereafter.

His endocrinology investigations included a normal short Synacthen test, thyroid-stimulating hormone (TSH), aldosterone-renin ratio, urinary metanephrines and repeat 9 am cortisol. Consequently, he was diagnosed with drug-induced hypoglycaemia secondary to tetracycline use. He was not tested for insulin or C-peptide levels because blood glucose levels were never recorded to be less than 2.2 mmol/L (Table 1).

**Table 1:** Investigations HbA1c, haemoglobin A1c; TSH, thyroid-stimulating hormone.

Investigation	Results	Reference
Post-prandial blood glucose	3.2 mmol/L	Under 7.8 mmol/L
Repeat post-prandial blood glucose	3.1 mmol/L	Under 7.8 mmol/L
HbA1c	33 mmol/mol	Below 42 mmol/mol
TSH	2.29 mU/L	0.30-4.20 mU/L
Aldosterone	157 pmol/L	Random sample upright: 90-720 pmol/L
Aldo/renin ratio	6 pmol/mU	
9 am cortisol	355 nmol/L	>350 nmol/L
Repeat 9 am cortisol	639 nmol/L	>350 nmol/L

Subsequently, the hypoglycaemic episodes resolved upon discontinuation of tetracycline.

This case presents a compelling illustration of the association between tetracycline use and hypoglycaemia in a non-diabetic adolescent. Throughout his medical journey, the patient exhibited no prior history of diabetes or related glycaemic disorders, rendering his hypoglycaemic episodes particularly noteworthy. Importantly, the absence of other concurrent medications or supplements during the course of tetracycline treatment emphasizes the distinctive role played by the antibiotic in inducing these episodes.

## Discussion

Hypoglycaemia, a relatively infrequent adverse event, has been linked to the use of tetracycline. The precise mechanism underlying this association remains uncertain, although previous reports have hinted at a potential interaction between tetracycline and glucose metabolism without providing a definitive understanding of the underlying pathophysiology [6]. In our case, the close temporal relationship between the initiation of tetracycline treatment, the onset of recurrent hypoglycaemic episodes, and the subsequent resolution of symptoms upon discontinuation strongly suggest a cause-and-effect relationship.

Nonetheless, the available evidence regarding the specific mechanisms involved remains limited. Existing reports on the hypoglycaemic effects of the antibiotics primarily involve patients who were also taking other medications [7], implying a potential interaction between these antibiotics and concomitant medications that may contribute to hypoglycaemic episodes. However, the occurrence of hypoglycaemia caused solely by these antibiotics, in the absence of other medications, is exceedingly rare.

The clinical significance of tetracycline-induced hypoglycaemia extends beyond the confines of these individual cases. It raises important questions about patient safety and prescription practices. Clinicians should be vigilant when prescribing tetracycline, especially in adolescents and young adults, and consider the potential for hypoglycaemic events even in those without prior glycaemic disorders. Given the rarity of this adverse event, awareness and recognition are paramount for timely intervention.

Further research is crucial to unravel the complex mechanisms at play and identify potential risk factors associated with tetracycline-induced hypoglycaemia. Comprehensive studies involving larger cohorts and controlled experiments are needed to shed light on the specific pathways through which tetracyclines may influence glucose metabolism. Such investigations will not only enhance our understanding of this intriguing phenomenon but also facilitate the development of guidelines for safer prescribing practices, ultimately minimizing the risk of hypoglycaemia in patients undergoing tetracycline therapy. In the absence of concrete evidence, it is incumbent upon healthcare providers to exercise caution, closely monitor patients, and consider alternative antibiotics when necessary to ensure the well-being of those under their care.

## Conclusions

Healthcare providers need to remain vigilant about the potential hazard of hypoglycaemia when prescribing tetracycline, especially in adolescent patients. Recognizing the symptoms promptly and discontinuing the medication can alleviate the symptoms and avert any potential complications. Further investigation is necessary to gain a comprehensive understanding of the underlying mechanisms and identify risk factors associated with hypoglycaemia induced by tetracycline. This knowledge will enable healthcare professionals to adopt safer and more informed prescribing practices.
